# Clinical and Biochemical Characterization of Fabry Disease Associated *GLA* Gene Variants: Data From a Large Cohort of 469 Thousand Genotyped Subjects of the UK Biobank Database

**DOI:** 10.1002/jimd.70103

**Published:** 2025-10-19

**Authors:** Antonina Giammanco, Carola Maria Gagliardo, Chiara Scrimali, Federica Brucato, Teresa Maria Grazia Fasciana, Maurizio Averna, Angelo Baldassare Cefalu, Davide Noto

**Affiliations:** ^1^ Regional Center for the Prevention Diagnosis and Cure of the Rare Metabolic Diseases (CERMMET) Sicily Italy; ^2^ Department of Health Promotion, Maternal and Child Health, Internal and Specialized Medicine of Excellence “G. D. Alessandro” (PROMISE) University of Palermo Palermo Italy; ^3^ Institute of Biophysics (IBF), National Research Council (CNR) Palermo Italy

**Keywords:** cardiovascular risk, Fabry disease, Fabry disease Fastex‐derived score, Fabry phenotype, FASTEX, pathogenetic variants

## Abstract

Fabry disease (FD) is a lysosomal storage disease due to genetic variants in the *GLA* gene located on the X chromosome. Males are hemizygous, while many females are genetic mosaics due to the random inactivation of the X chromosome. While most of the identified variants are deleterious for *GLA*, in some cases, less rare gene variants have been considered responsible for some FD features. *GLA* variants were selected from the database of 469 thousand genotyped subjects of the UK Biobank database. Pathogenic variants (ALL_P), variants of uncertain significance (ALL_U), and variants with conflicting interpretations of pathogenicity (ALL_C) were grouped, while p.Asp313Tyr, p.Ala143Thr, p.Ser126Gly, p.Arg118Cys, and p.Asn215Ser were evaluated individually. More than 480 thousand subjects not carrying variants in the *GLA* gene were used as controls in association studies. Clinical and biochemical phenotypes were extracted from the same database, and a FD phenotype score (FASTEX derived Fabry, FDF score) was derived from the FASTEX prognostic to assess the probability of an FD phenotype score. Pathogenic variants and p.Asn215Ser were associated with FDF score, while all other variants were not associated with any FDF feature. Stratification of patients based on a calculated cardiovascular (CV) risk score demonstrated that patients with nonpathogenic variants within the highest CV risk quartile (> 75th percentile) showed characteristic features of FD, whereas those in the lower risk group (< 75th percentile) showed odds ratios indicating inverse association with FD features. In conclusion, the data from UK Biobank suggest that pathogenic variants are always associated with FD features, while variants of uncertain significance and conflicting interpretation acquire an FD phenotype only in the presence of a high CV risk burden.

## 1 Introduction

Fabry Disease (FD, OMIM #301500) is a lysosomal storage disease caused by disruptive variants in the *GLA* gene [1]. *GLA* is located on the X chromosome and encodes the lysosomal alpha galactosidase A (AGA) enzyme. AGA produces glucosyl‐ceramide from galactosyl‐ceramide by detaching a pentose unit from the sugar chain. The disruption of the pathway leads to the accumulation of different glycosphingolipid (GSL) species, namely globotriaosylceramide (GB3), which is converted into LysoGB3 by acid ceramidase. LysoGB3 is the biomarker of GSL accumulation in FD and is commonly used as the diagnostic biomarker of the disease [2]. FD is an X‐linked disease: males are affected since hemizygous while females may present a broad spectrum of phenotypes, from asymptomatic to severe multi‐organ involvement. In addition, females harboring the same GLA variant may be affected in different organs with different degrees. This variability could be partially explained by the dynamic and complex process of random X chromosome inactivation in different tissues [2, 3].

The genetic heterogeneity of the disease is also complicated by the different effects of *GLA* variants on the AGA structure. The effects of the variants on *GLA* influence AGA residual enzymatic activity and classify FD into “classic” and “non‐classic” (or “late‐onset”) forms. Mainly, the classic FD phenotype is associated with *GLA* variants determining absent or severely reduced AGA activity, early onset of the disease, and multiple organ involvement. Other variants determining residual AGA activity are responsible for the non‐classic FD phenotype, characterized by tardive clinical manifestations and confined organ involvement (predominant cardiac subtype) [4]. Moreover, many other variants display unclear clinical phenotypes and are classified as variants of uncertain significance (VUS) [4]. Determining the pathogenicity of these variants remains challenging since FD clinical evidence of the disease starts when enough GSL molecules accumulate in the body, usually in childhood. GSL accumulation leads to metabolic dysfunctions and inflammatory events, with progressive organ dysfunction. First child manifestations usually include gastrointestinal (diarrhea or alternating constipation) and neuropathic symptoms (from mild sweating to anhidrosis) [5]. Nevertheless, the deleterious outcomes, mainly heart and kidney impairments, emerge later in life and progress rapidly, leading to early organ failure [1, 2]. Some *GLA* variants show an elevated allele frequency in the general population (see Table 1), and their association with FD may remain uncertain because the main FD clinical features, such as left ventricular hypertrophy, renal insufficiency, and cerebral stroke, occur in adult age and may be attributable to conventional cardiovascular (CV) risk factors. FD cannot be distinguished in these cases from an FD phenocopy due, as an example, to hypertensive cardiomyopathy with secondary hypertensive renal impairment and stroke [6]. The risk of a false attribution of a causative role for a *GLA* variant derives from the selection bias of study designs published so far, which researched *GLA* variants only in subjects with a suggestive FD phenotype, while healthy controls (patients without signs of FD) were not genotyped for comparison.

**TABLE 1:** *GLA* variants groups and relative sample numerosity.

Variants	Hemizygous male	Wild type male	Heterozygous female	Wild type female
Conflicting interpretation (ALL_C)	1587 (0.0075)	210 097 (0.9925)	3833 (0.01507)	250 469 (0.98485)
Pathogenic (ALL_P)	31 (0.00014)	214 046 (0.99986)	44 (0.00017)	254 422 (0.99983)
VUS (ALL_U)	106 (0.00050)	211 043 (0.9995)	181 (0.00071)	254 141 (0.99929)
p.Asp313Tyr_C	854 (0.00397)	214 197 (0.99603)	1990 (0.00782)	252 492 (0.99216)
p.Arg118Cys_C	172 (0.00080)	214 813 (0.9992)	441 (0.00173)	254 048 (0.99827)
p.Ser126Gly_C	167 (0.00078)	214 835 (0.99922)	328 (0.00129)	254 159 (0.99871)
p.Ala143Thr_C	116 (0.00054)	214 894 (0.99946)	317 (0.00125)	254 171 (0.99875)
p.Asn215Ser_P	13 (0.000059)	214 864 (0.99994)	31 (0.00012)	254 448 (0.99988)

*Note:* Data are expressed as raw number of variants carriers and proportions relative to the same gender in brackets. Suffixes (_C, _P) indicate conflicting interpretation and pathogenic variants, respectively.

This manuscript presents a different approach by leveraging a large dataset of over 469 thousand subjects genotyped for *GLA* variants in the UK Biobank project [7], thereby association of FD features with variants can be established with precision due to the large number of healthy controls available. The large database of clinical features of the biobank also allowed for the evaluation of the contribution of common cardiovascular (CV) risk factors and biochemical variables to the association of *GLA* variants with FD features.

## 2 Methods

### 2.1 Study Sample

The study was conducted on the cohort of the UK Biobank considering only the subjects with available whole exome sequencing (WES) data. The final cohort consisted of 469 563 subjects (254 489 females, 215 074 males) from the uk23518 final release of exome data. The study utilized several clinical, biochemical, and genetic variables, each with varying degrees of missing data. The coverage of these variables across the entire cohort is illustrated in Figure S1.

### 2.2 *GLA* Variants

*GLA* gene variants were extracted from the exome data using the GRCh38 genomic coordinates of the *GLA* gene (X:101.397.803–101.407.925) using Plink 2.0 within the online DNAnexus Research Analysis Platform (RAP). A total of 477 variants were extracted. Variants were coded according to the Clinical Significance field of the opencravat annotation file as: pathogenic (*n* = 9), conflicting classification (*n* = 28), uncertain significance (*n* = 43) variants. Variants with a minor allele frequency (MAF) sufficiently high to allow statistical calculation were also analyzed individually; these included p.Asp313Tyr, p.Arg118Cys, p.Ser126Gly, p.Ala143Thr (conflicting variants) and p.Asn215Ser (pathogenic variant). All the benign, likely benign, likely pathogenic, or not annotated variants were excluded from the study. A set of suffixes: _C, _P, and _U was added to the variant name to indicate the relative group of variants: conflicting, pathogenic, uncertain significance (VUS) respectively. The list of the main investigated variants and groups is presented in Table 1, while the complete list of the investigated *GLA* variants is presented in Table S1.

### 2.3 Fastex Derived Fabry (FDF) Score

Clinical domains of FD were extracted from the “41270” field of the UK Biobank database, which stores the subject's clinical conditions using the ICD10 coding system. Certain numerical variables were also used to build the “Fastex Derived Fabry (FDF) Score” and were extracted as individual fields from the UK Biobank database. A clinical score of FD features (FDF Score) was built using items and scoring criteria from the published FASTEX scoring system [8]. The pain component was assessed by the “pain” questionnaire included in the Biobank database. Since the pain domain contained a significant amount of missing data, the lower category of pain (0 points) was inferred for missing items. It is to note that the FDF Score is “derived” from FASTEX, which is a scoring system designed to monitor FD progression rather than for diagnostic purposes. In our setting, the FDF score was used to assess the probability of an FD phenotype in the UK Biobank population. The comparison of the two scores and the derivation of the FDF score are summarized in Table S2.

### 2.4 Statistics

Participants were stratified into four cohorts based on *GLA* gene variant classification. Within the UK Biobank dataset, individuals were categorized according to the presence of pathogenic variants (ALL_P), variants with conflicting interpretations (ALL_C), or variants of uncertain significance (ALL_U). Genotyped individuals lacking *GLA* variants served as wild‐type controls. To allow valid sex‐matched comparisons, homozygous female were excluded: hemizygous males were compared to male controls, and heterozygous females to female controls. This filtering strategy mitigated sex imbalance within the hemizygous group. As a result, 19 homozygous females carrying variants of conflicting interpretation were excluded; no homozygous females with pathogenic or uncertain variants were identified.

The FDF score was dichotomized at a threshold of > 4 versus ≤ 4. This cutoff ensured that scores above 4 reflected the involvement of at least two clinical domains, given the domain‐specific maximum score of 4. The appropriateness of this threshold was supported by receiver operating characteristic (ROC) analysis, where the optimal cutoff for predicting pathogenic variant carriers (ALL_P group), based on the Youden index, was 4.4 (data not shown).

Associations between *GLA* variants—both individually and in groups—and the dichotomized FDF score, as well as each clinical domain, were assessed using automated ordinal logistic regression models via the “polr” function in R. All models were adjusted for major cardiovascular and renal risk factors, including age, diabetes mellitus, dyslipidemia, hypertension, obesity, and smoking status. Risk factors were derived from the UK Biobank field “41270”, while pain‐related variables were obtained from the Biobank's pain questionnaire (Table S2). Binary outcomes were modeled using logistic regression (R glm function, family = “binomial”). The R glm function accounted for the missing data, excluding observations with one or more missing data in the dependent variable or predictors.

Associations between *GLA* variants and continuous phenotypes were evaluated using multiple linear regression models, with the same covariate set. To correct for multiple comparisons, a custom routine implementing the Benjamini–Hochberg procedure was applied to the full set of *p*‐values. All continuous predictors were modeled linearly, without transformation or inclusion of polynomial terms. Multicollinearity was assessed a priori using variance inflation factors (VIFs) from the “faraway” R package; all models met acceptable collinearity thresholds.

All data processing and analyses were conducted using the RStudio environment (a graphical interface for R), executed either locally or within the UK Biobank Research Analysis Platform (RAP). Previously published automated scripts were employed to perform high‐throughput regression modeling [9]. All computations and custom pipelines were implemented in R within RStudio for Windows; selected routines were run in Jupyter notebooks within RAP, using a combination of Bash and Python.

## 3 Results

**FIGURE 1:**
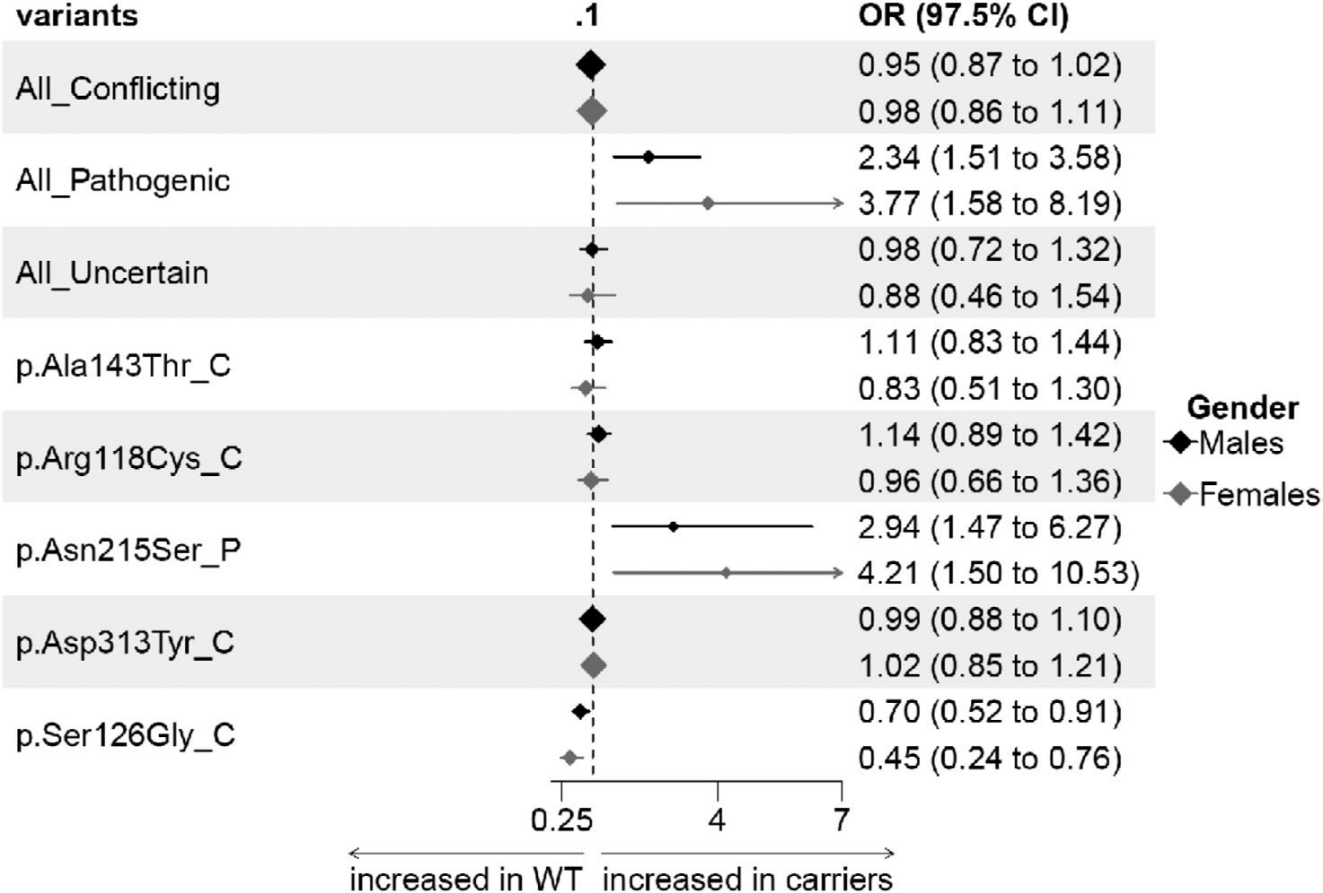
Association between *GLA* variants and FDF score (> 4 points). Data are expressed as Odds Ratio and 97.5% confidence intervals in brackets. OR were adjusted for CV risk factors: age, diabetes mellitus, dyslipidemia, hypertension, smoking, and obesity. The suffixes in individual variants are: _C = variants with conflicting interpretation of pathogenicity, _P = pathogenic variant. Indicators are proportional to the number of carriers (log transformed).

The association of *GLA* variants with FD phenotype, expressed as FDF score > 4, is presented in Figure 1. *GLA* variants were either grouped according to variant classification (conflicting, VUS, pathogenic) or presented individually for the more frequent variants (p.Asp313Tyr, p.Arg118Cys, p.Ser126Gly, p.Ala143Thr, and p.Asn215Ser). Odds ratios were adjusted for the main CV risk factors. The figure shows that only pathogenic variants, both ALL_P and p.Asn215Ser alone, associated significantly with the FD phenotype, while all other variants were not associated after correction for the common CV risk factors. The p.Ser126Gly variant even showed a paradoxical protective effect with a significantly reduced odds ratio both in males and females.

Individual clinical domains of the FDF score were also tested separately for their association with the *GLA* variants. The relative results are presented in Figure S2A–F. Data regarding the FDF “pain” item are not shown because of the low numerosity of patients reporting pain in the “pain questionnaire”. Figure S1 confirmed the findings of the composite FDF score, except for the VUS male carriers that were associated with the estimated glomerular filtration rate (eGFR) component of the renal domain of the FDF score with an Odds Ratio of 1.66 (1.25–2.15), and for the association of the heterozygous female for the p.Arg118Cys variant with the urinary Albumin to Creatinine ratio (uACR) component of the renal domain of the FDF score, with an Odds Ratio of 1.24 (1.02–1.50), showing that also nonpathogenic variants might have some deleterious effect on renal function. The data regarding numerical variables are presented in Table 2A (males) and Table 2B (females). Data are expressed as mean ± standard deviation and relative MRA multiple testing adjusted *p*‐values.

**TABLE 2A:** Numerical parameters ratios of mean values in male hemizygous vs. male controls.

Variable	Controls	ALL conflicting	ALL_Pathogenic	ALL_Uncertain	p.Asp313Tyr_C	p.Arg118Cys_C	p.Ser126Gly_C	p.Ala143Thr_C	p.Asn215Ser_P
Clinical									
BMI (kg/m^2^)	27.834 ± 4.21	27.647 ± 4.20 (0.328)	28.219 ± 4.28 (0.621)	27.389 ± 4.97 (0.641)	27.532 ± 4.21 (0.294)	27.525 ± 3.84 (0.940)	27.67 ± 4.26 (0.660)	27.615 ± 4.15 (0.810)	29.046 ± 5.13 (0.849)
Systolic BP (mm/Hg)	142.706 ± 18.47	142.734 ± 18.87 (0.799)	142.586 ± 15.87 (0.890)	144.388 ± 21.09 (0.643)	142.549 ± 18.31 (0.43)	143.552 ± 20.11 (0.940)	143.558 ± 19.75 (0.841)	140.491 ± 20.90 (0.699)	147.833 ± 18.12 (0.717)
Diastolic BP (mm/Hg)	84.005 ± 10.54	83.627 ± 10.96 (0.328)	83.828 ± 8.24 (0.746)	85.049 ± 11.17 (0.643)	83.451 ± 10.94 (0.294)	84.006 ± 10.77 (0.940)	83.135 ± 10.75 (0.573)	82.173 ± 12.49 (0.699)	86.417 ± 10.24 (0.849)
EKG									
PR interval (ms)	170.89 ± 28.97	173.56 ± 31.93 (0.712)	172 ± 5.66 (0.881)	179.27 ± 43.9 (0.774)	174.1 ± 33.88 (0.796)	166.18 ± 31.36 (0.881)	168.33 ± 26.02 (0.878)	178.83 ± 45.63 (0.351)	176.12 ± NA [1] (NA)
QTc interval (ms)	416.43 ± 25.07	418.86 ± 27.81 (0.701)	453.5 ± 4.95 (0.521)	423.58 ± 18.87 (0.736)	420.24 ± 29.36 (0.661)	413.5 ± 17.73 (0.931)	417.77 ± 30.14 (0.966)	410.58 ± 27.4 (0.426)	457.00 ± NA [1] (NA)
Biochemical									
Urea (mmol/L)	5.613 ± 1.45	5.544 ± 1.33 (0.328)	6.133 ± 1.45 (0.255)	5.565 ± 1.30 (0.843)	5.557 ± 1.37 (0.572)	5.652 ± 1.26 (0.940)	5.487 ± 1.22 (0.573)	5.546 ± 1.35 (0.810)	5.839 ± 1.04 (0.863)
Creatinine (mmol/L)	81.745 ± 19.10	80.765 ± 12.97 (0.328)	85.829 ± 16.84 (0.616)	80.459 ± 12.52 (0.843)	80.955 ± 13.2 (0.572)	80.531 ± 12.75 (0.940)	79.488 ± 11.15 (0.573)	80.554 ± 14.45 (0.810)	83.108 ± 15.1 (0.936)
Cystatin_C (mg/L)	0.943 ± 0.18	0.938 ± 0.15 (0.328)	0.95 ± 0.18 (0.832)	0.922 ± 0.12 (0.643)	0.936 ± 0.15 (0.572)	0.941 ± 0.14 (0.972)	0.941 ± 0.14 (0.573)	0.925 ± 0.16 (0.810)	1.003 ± 0.22 (0.789)
eGFR (mL/min)	100.58 ± 20.32	101.18 ± 19.92 (0.328)	97.53 ± 22.97 (0.491)	101.94 ± 20.29 (0.843)	101.03 ± 20.03 (0.632)	101.44 ± 20.25 (0.754)	101.74 ± 18.98 (0.573)	101.78 ± 20.55 (0.810)	100.31 ± 23.97 (0.863)
U Creatinine (μmol/L)	10 881 ± 6116	10 813 ± 6149 (0.873)	10 108 ± 5049 (0.616)	10 879 ± 6855 (0.937)	10 911 ± 6256 (0.899)	10 707 ± 6721 (0.940)	11 082 ± 6253 (0.832)	9972 ± 4973 (0.699)	10 793 ± 5904 (0.863)
uACR (mg/g)	20.527 ± 43.21	20.095 ± 39.42 (0.928)	**84.301 ± 119.2 (< 0.0001)**	23.567 ± 29.73 (0.843)	18.516 ± 31.32 (0.567)	15.02 ± 16.72 (0.940)	23.163 ± 59.47 (0.832)	13.563 ± 11.54 (0.810)	**76.491 ± 79.59 (0.015)**
U uAlbumin (mg/L)	37.028 ± 152.25	37.961 ± 157.5 (0.873)	65.176 ± 66.00 (0.661)	22.759 ± 23.13 (0.843)	37.831 ± 172.88 (0.785)	20.891 ± 27.35 (0.940)	44.716 ± 132.40 (0.832)	16.32 ± 13.94 (0.810)	66.171 ± 60.23 (0.863)
Glucose (mmol/L)	5.189 ± 1.40	5.169 ± 1.18 (0.873)	5.048 ± 0.76 (0.746)	5.361 ± 1.76 (0.843)	5.172 ± 1.20 (0.899)	4.965 ± 0.68 (0.530)	5.243 ± 1.07 (0.832)	5.122 ± 0.97 (0.810)	4.71 ± 0.63 (0.484)
HbA1c (mmol/mol)	36.515 ± 7.60	36.249 ± 6.94 (0.328)	34.487 ± 3.8 (0.491)	37.655 ± 9.54 (0.643)	36.121 ± 6.32 (0.632)	35.078 ± 4.75 (0.400)	37.502 ± 8.47 (0.832)	35.73 ± 6.24 (0.810)	35.415 ± 3.92 (0.484)
Urate (μmol/L)	354.62 ± 71.748	351.46 ± 71.56 (0.328)	382.00 ± 78.57 (0.491)	347.47 ± 69.478 (0.643)	348.70 ± 70.106 (0.294)	355.14 ± 75.544 (0.940)	346.59 ± 67.99 (0.573)	356.55 ± 85.775 (0.930)	392.23 ± 97.956 (0.484)
Lipids									
Cholesterol (mmol/L)	5.479 ± 1.13	5.495 ± 1.097 (0.928)	5.242 ± 0.975 (0.616)	5.591 ± 1.176 (0.643)	5.504 ± 1.11 (0.899)	5.372 ± 0.974 (0.326)	5.557 ± 1.07 (0.573)	5.468 ± 1.028 (0.930)	5.22 ± 1.254 (0.863)
Triglycerides (mmol/L)	1.98 ± 1.155	1.945 ± 1.162 (0.491)	1.867 ± 1.063 (0.616)	1.987 ± 1.072 (0.843)	1.901 ± 1.129 (0.294)	1.975 ± 1.194 (0.836)	1.903 ± 1.048 (0.573)	1.987 ± 1.3 (0.930)	1.76 ± 1.107 (0.484)
HDL chol (mmol/L)	1.279 ± 0.312	1.293 ± 0.317 (0.328)	1.296 ± 0.409 (0.616)	1.317 ± 0.296 (0.643)	1.305 ± 0.31 (0.294)	1.304 ± 0.336 (0.940)	1.322 ± 0.341 (0.573)	1.265 ± 0.337 (0.810)	1.441 ± 0.555 (0.169)
ApoB (g/L)	1.026 ± 0.239	1.021 ± 0.231 (0.328)	0.982 ± 0.22 (0.616)	1.051 ± 0.245 (0.643)	1.021 ± 0.237 (0.572)	0.987 ± 0.218 (0.089)	1.034 ± 0.231 (0.832)	1.025 ± 0.213 (0.930)	0.962 ± 0.272 (0.849)
Lipoprotein(a) (nmol/L)	43.855 ± 49.02	44.655 ± 49.98 (0.328)	54.805 ± 54.78 (0.616)	42.109 ± 40.76 (0.843)	43.446 ± 49.87 (0.899)	45.93 ± 48.72 (0.940)	45.555 ± 53.23 (0.832)	47.134 ± 51.40 (0.810)	72.742 ± 54.29 (0.484)

*Note:* Data are expressed as hemi *GLA* carriers/wt means ratio (fold) with multiple regression analysis *p*‐value in brackets. MRA are adjusted for age, diabetes mellitus, hypertension, dyslipidemia, obesity, and smoking. Lipids data were also adjusted for statins therapy. Multiple tests adjustments were performed by Benjamini‐Hochberg (BH) procedure. Suffixes (_C, _P, _U) indicate conflicting interpretation, pathogenic, and uncertain significance variants, respectively [1]. There was only one mutated subject, then *p* value and standard deviation were not calculated. Bold values indicate *p* values < 0.05.

**TABLE 2B:** Numerical parameters ratios of mean values in female heterozygous vs. female controls.

Variable	Controls	ALL_Conflicting	ALL_Pathogenic	ALL_Uncertain	p.Asp313Tyr_C	p.Arg118Cys_C	p.Ser126Gly_C	p.Ala143Thr_C	p.Asn215Ser_P
Clinical									
BMI (kg/m^2^)	27.075 ± 5.14	26.926 ± 5.075 (0.06)	25.827 ± 5.71 (0.258)	26.557 ± 4.69 (0.511)	26.806 ± 5.079 (0.119)	27.18 ± 5.22 (0.927)	26.736 ± 4.61 (0.746)	27.162 ± 5.38 (0.848)	25.913 ± 5.83 (0.655)
Systolic BP (mm/Hg)	137.197 ± 20.22	137.86 ± 20.54 (0.271)	**126.81 ± 18.00 (< 0.002)**	140.605 ± 21.74 (0.504)	137.454 ± 20.55 (0.893)	139.036 ± 20.40 (0.592)	137.785 ± 20.92 (0.994)	139.542 ± 21.27 (0.325)	**125.448 ± 18.36 (0.010)**
Diastolic BP (mm/Hg)	80.677 ± 10.556	80.715 ± 10.297 (0.945)	77.905 ± 10.00 (0.258)	82.058 ± 11.67 (0.504)	80.693 ± 10.28 (0.893)	81.04 ± 10.03 (0.927)	80.797 ± 10.68 (0.994)	81.111 ± 10.05 (0.580)	75.966 ± 10.43 (0.142)
EKG									
PR interval (ms)	160.17 ± 24.80	160.18 ± 24.8 (0.939)	160.19 ± 24.81 (0.890)	160.18 ± 24.82 (0.821)	160.17 ± 24.79 (0.824)	160.19 ± 24.81 (0.890)	160.19 ± 24.82 (0.799)	160.19 ± 24.81 (0.225)	160.19 ± 24.81 (0.625)
QTc interval (ms)	426.61 ± 25.34	426.61 ± 25.35 (0.444)	426.65 ± 25.3 (0.890)	426.66 ± 25.3 (0.793)	426.62 ± 25.31 (0.444)	426.64 ± 25.31 (0.631)	426.64 ± 25.29 (0.656)	426.64 ± 25.31 (0.336)	426.65 ± 25.3 (0.372)
Biochemical									
Urea (mmol/L)	5.23 ± 1.33	5.265 ± 1.35 (0.479)	5.405 ± 1.81 (0.497)	5.128 ± 1.22 (0.508)	5.301 ± 1.40 (0.172)	5.177 ± 1.25 (0.828)	5.247 ± 1.32 (0.994)	5.302 ± 1.29 (0.555)	5.289 ± 1.69 (0.975)
Creatinine (mmol/L)	64.373 ± 13.79	64.667 ± 12.11 (0.458)	**73.637 ± 70.96 (< 0.0001)**	63.836 ± 10.76 (0.824)	64.71 ± 12.08 (0.570)	64.406 ± 12.25 (0.991)	64.343 ± 11.57 (0.994)	65.041 ± 10.76 (0.555)	62.914 ± 9.74 (0.904)
Cystatin_C (mg/L)	0.878 ± 0.167	0.879 ± 0.15 (0.807)	**0.991 ± 0.86 (< 0.0001)**	0.853 ± 0.13 (0.412)	0.884 ± 0.16 (0.438)	0.877 ± 0.16 (0.927)	0.876 ± 0.14 (0.994)	0.871 ± 0.13 (0.549)	0.867 ± 0.18 (0.904)
eGFR (mL/min)	100.438 ± 20.42	99.876 ± 20.86 (0.458)	99.949 ± 23.33 (0.861)	100.542 ± 18.81 (0.824)	99.615 ± 20.44 (0.438)	101.136 ± 22.76 (0.869)	100.456 ± 21.29 (0.994)	98.608 ± 20.08 (0.549)	101.797 ± 19.85 (0.904)
U Creatinine (μmol/L)	7186 ± 4953	7000 ± 4791 (0.067)	7504 ± 4725 (0.861)	6993 ± 4798 (0.824)	6911 ± 4715 (0.119)	7231 ± 5122 (0.991)	7261 ± 5284 (0.994)	6849 ± 4477 (0.549)	7032 ± 4701 (0.975)
uACR (mg/g)	20.993 ± 38.42	20.196 ± 34.71 (0.697)	22.359 ± 21.38 (0.918)	18.362 ± 16.22 (0.824)	20.354 ± 38.79 (0.893)	22.273 ± 38.07 (0.927)	16.781 ± 23.58 (0.994)	17.119 ± 19.99 (0.549)	23.2 ± 22.75 (0.975)
U uAlbumin (mg/L)	24.444 ± 95.71	26.127 ± 117.06 (0.697)	64.548 ± 190.29 (0.255)	16.985 ± 13.64 (0.824)	33.057 ± 160.09 (0.162)	17.001 ± 17.73 (0.669)	17.785 ± 23.18 (0.994)	14.68 ± 10.60 (0.549)	83.847 ± 234.70 (0.142)
Glucose (mmol/L)	5.068 ± 1.07	5.071 ± 1.07 (0.909)	5.284 ± 1.25 (0.497)	5.088 ± 1.29 (0.901)	5.073 ± 1.03 (0.893)	5.144 ± 1.15 (0.669)	5.195 ± 1.75 (0.582)	5.016 ± 0.75 (0.555)	5.427 ± 1.49 (0.628)
HbA1c (mmol/mol)	35.807 ± 5.97	35.707 ± 5.73 (0.240)	35.577 ± 4.26 (0.429)	36.651 ± 7.93 (0.508)	35.668 ± 5.49 (0.172)	35.661 ± 6.47 (0.592)	36.046 ± 6.96 (0.994)	35.477 ± 4.97 (0.549)	35.686 ± 4.56 (0.591)
Urate (μmol/L)	271.06 ± 66.27	269.159 ± 64.73 (0.065)	261.209 ± 78.49 (0.497)	263.658 ± 62.39 (0.504)	268.623 ± 65.50 (0.136)	268.445 ± 59.96 (0.592)	266.772 ± 65.19 (0.746)	269.85 ± 60.83 (0.733)	261.345 ± 83.82 (0.683)
Lipids									
Cholesterol (mmol/L)	5.874 ± 1.12	5.856 ± 1.11 (0.340)	5.960 ± 1.35 (0.861)	5.850 ± 1.12 (0.901)	5.874 ± 1.13 (0.896)	5.795 ± 1.04 (0.592)	5.898 ± 1.17 (0.994)	5.854 ± 1.12 (0.549)	5.972 ± 1.41 (0.975)
Triglycerides (mmol/L)	1.554 ± 0.86	**1.511 ± 0.79 (0.001)**	1.590 ± 1.21 (0.961)	1.503 ± 0.73 (0.824)	1.530 ± 0.80 (0.172)	1.551 ± 0.84 (0.869)	1.532 ± 0.83 (0.994)	1.444 ± 0.71 (0.152)	1.62 ± 1.16 (0.975)
HDL chol (mmol/L)	1.593 ± 0.37	**1.619 ± 0.38 (< 0.0005)**	1.677 ± 0.32 (0.395)	1.584 ± 0.37 (0.824)	1.614 ± 0.38 (0.119)	1.608 ± 0.39 (0.592)	1.633 ± 0.38 (0.582)	1.65 ± 0.40 (0.152)	1.683 ± 0.33 (0.655)
ApoB (g/L)	1.037 ± 0.23	**1.028 ± 0.23 (0.028)**	1.031 ± 0.28 (0.861)	1.033 ± 0.24 (0.950)	1.031 ± 0.23 (0.344)	1.018 ± 0.22 (0.523)	1.04 ± 0.24 (0.994)	1.031 ± 0.22 (0.549)	1.038 ± 0.28 (0.905)
Lipoprotein(a) (nmol/L)	45.279 ± 49.37	45.029 ± 49.06 (0.807)	31.533 ± 36.26 (0.285)	40.824 ± 46.22 (0.637)	45.254 ± 48.91 (0.949)	43.575 ± 49.97 (0.869)	47.771 ± 51.99 (0.46)	40.025 ± 49.27 (0.395)	32.395 ± 38.51 (0.628)

*Note:* Bold values indicate *p* values < 0.05.

The carriers of pathogenic variants showed increased uACR, but not eGFR. Renal damage parameters were also increased in heterozygous females with pathogenic variants. Females exhibiting conflicting interpretation of *GLA* variants showed increased HDL cholesterol and reduced triglyceride and apolipoprotein B levels compared to controls.

**FIGURE 2:**
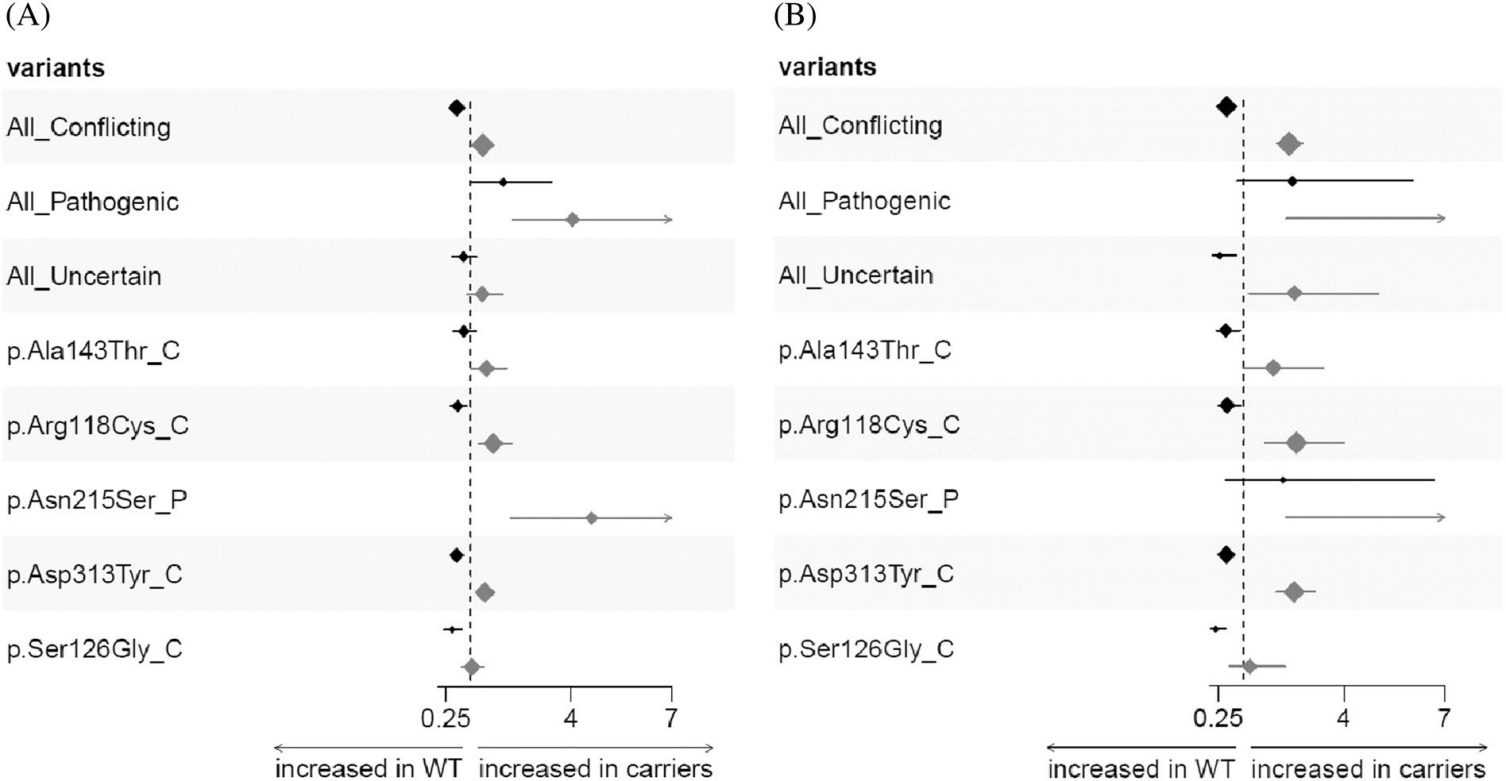
Association between *GLA* variants and FDF score (> 4 points) according to the CV risk score. (A) Male subjects. (B) Female subjects. Data are expressed as Odds Ratio and 97.5% confidence intervals in brackets. OR were adjusted for CV risk factors: age, diabetes mellitus, dyslipidemia, hypertension, smoking, and obesity. The suffixes in individual variants are: _C = variants with conflicting interpretation of pathogenicity, _P = pathogenic variant. Indicators are proportional to the number of carriers (log transformed).

The contribution of the CV risk factors to the association of *GLA* variants with the FD phenotype, expressed by the FDF score, was evaluated by calculating a CV risk score using a logistic regression model as shown in Table S3. The goodness of the model was assessed by its power to predict the presence of a pathogenic *GLA* variant as shown in the ROC curve analysis of Figure S3. The CV risk score predicted the presence of GLA pathogenic variants with an AUC of 0.80, sensitivity = 0.77, and specificity = 0.70 for a threshold of CV score = 1.92. The association of *GLA* variants with the FD phenotype was then evaluated separately in patients with a high CV risk score (> 75 percentile) and with a low CV risk score (< 75 percentile), both compared with the reference population. Consequently, the logistic regressions were not CV risk adjusted in this set of analyses. The results are presented in Figure 2 panel A (males) and panel B (females). The results show that the contribution of CV risk factors to the FD phenotype is additive but not relevant for both hemizygous males and heterozygous females with pathogenic *GLA* variants. Conversely, the carriers of VUS or conflicting interpretation variants are associated with an FD phenotype only in the presence of an elevated CV risk score, while lower CV risk scores (< the 75% percentile) associated VUS or conflicting interpretation *GLA* variants with a significantly reduced risk of an FD phenotype.

## 4 Discussion

FD is a rare metabolic disease characterized by clinical involvement of different organs and tissues due to the accumulation of sphingolipids that are not correctly catabolized by the dysfunctional AGA enzyme. The FD clinical phenotype is heterogeneous and ambiguous, meaning that FD clinical manifestations resemble common diseases, such as chronic kidney disease, cardiovascular disease with hypertrophic cardiomyopathy, psychiatric disorders, generalized pain in early stages, and gastrointestinal disturbances. Hearing loss, cornea verticillata, and skin angiokeratomas are often present. The role of *GLA* variants as a cause of FD can be elusive in females, in whom the X chromosome mosaicism alters the clinical presentation, as well as in males and females carrying variants whose pathogenicity cannot be established with certainty, and variants with conflicting interpretations of pathogenicity or VUS [3, 4]. Many of these variants were previously considered pathogenic, only to be declassified as non‐pathogenic later on. The main limitation in assessing a causative role for these variants lies in the selection bias of studies investigating gene variants in cohorts of subjects with the FD phenotype. Nevertheless, the co‐existence of a *GLA* variant with an FD‐like phenotype does not establish a cause–effect relationship in the presence of co‐expressed common risk factors. The use of the UK biobank, with hundreds of thousands of wild‐type unaffected genotyped individuals, prevented this kind of selection bias.

Using this population approach, we found that pathogenic *GLA* variants were effectively associated with a FD phenotype, namely the FDF score of the present study, and that VUS or conflicting interpretation variants were associated neither with the FD phenotype nor with the individual FD features, as shown in Figure 1 and Tables 2A and 2B. Using a logistic regression model to calculate a CV risk score that included the main CV risk factors and stratified subjects according to the *GLA* variants in high and low CV risk, we showed that only *GLA* pathogenic variants are associated with the FD phenotype irrespective of the coexistence of an elevated CV risk. On the contrary, uncertain significance or conflicting interpretation variants were associated with the FD phenotype only in subjects with an elevated CV risk (Figure 2A,B).

This finding suggests that many *GLA* non‐pathogenic variants were considered causative of FD in the past because of the coexistence of common CV risk factors in the selected patients.

Due to the large number of genotyped subjects, we were able to analyze individually the most frequent *GLA* variants, some of which have been extensively debated in recent literature. We discussed these variants individually below.

### 4.1 *GLA* P.Asp313Tyr (D313Y)

This variant has been widely investigated. Cardiac MR (CMR) did not show heart alterations by Late Gadolinium Enhancement (LGE), T1 mapping, or CMR‐feature tracking myocardial strain [10]. A polyneuropathic phenotype was confirmed by analysis of small skin fibers in 7 out of 9 carriers [11]. CKD, signs of previous stroke, white matter lesion in brain MR, and polyneuropathy were found in other collections of p.Asp313Tyr carriers. However, in other cases, no features consistent with FD were identified, and LysoGb3 values remained below the diagnostic threshold in all patients evaluated in a previous study [12]. Another report described four females with cerebrovascular events, seven with pain or acroparesthesia, and one with cornea verticillata; Lyso‐Gb3 was elevated in two females with brain involvement [13]. The present analysis regarding 859 hemizygous males and 1990 heterozygous females did not reveal any association of this variant with the FD phenotype (Figure 1 and Tables 2A and 2B) or with any of the FD clinical domains (Figure S2A–F), suggesting that p.Asp313Tyr should not be considered causative of non‐classic FD. A recent meta‐analysis reached the same conclusion [14].

### 4.2 *GLA* P.Ala143Thr (A143T)

The pathogenic role of this variant has been questioned [15]. As for the other variants, most of the reported cases derive from collections of patients affected by cardiomyopathies, chronic kidney disease (CKD), or stroke, lacking comparison with the background healthy populations [16]. Cardiomyopathy was detected in three affected members of a family harboring the variant [17], while no abnormal CMR by LGE, T1 mapping, or CMR‐feature tracking myocardial strain were found in p.Ala143Thr carriers [10]. A further study suggested a potential association with cardiac phenotypes, despite normal LysoGb3 levels [18]. Human skin fibroblasts (HSF) showed reduced enzyme activity but not LysoGB3 accumulation [19]. The present data, regarding 116 hemizygous males and 317 heterozygous females, did not show any association of this variant with the FD phenotype (Figure 1 and Tables 2A and 2B) or with any of the FD clinical domains (Figure S2A–F), suggesting that also p.Ala143Thr should not be considered causative of non‐classic FD.

### 4.3 *GLA* P.Ser126Gly (S126G)

The variant has been investigated as a potential cause of nonclassic FD. Different reports suggested that the p.Ser126Gly variant might not be related to FD, both in vivo [19, 20] and in vitro, considering cells derived from variant carriers, such as HSF, induced pluripotent stem cells (iPSC), and iPSC‐derived sensory neurons [19, 21]. Endoplasmic Reticulum (ER) stress has been detected in Human Embryonic Kidney (HEK) 293 cells and COS‐7 cells expressing the p.Ser126Gly *GLA* variant [22]. The present data, including 167 hemizygous males and 238 heterozygous females (Figure 1 and Figure S2A–F), indicated a paradoxical protective effect of this variant in both genders, with a negative trend for all the FDF score items. This variant showed the weakest evidence of association with the FD phenotype among those presented in this study.

### 4.4 *GLA* P.Arg118Cys (R118C)

Elevated LysoGB3 levels [23] and sporadic observations of FD phenotypes [24] have suggested that p.Arg118Cys might be causative of non‐classic FD, but this claim has not been confirmed in other studies [25]. The present data, regarding 172 hemizygous males and 441 heterozygous females, did not show any association of this variant with the FD phenotype (Figure 1 and Tables 2A and 2B) or any of the FD clinical domains (Figure S2A–F), suggesting that p.Arg118Cys should likewise not be considered causative of non‐classic FD.

### 4.5 *GLA* P.Asn215Ser (N215S)

This pathogenic variant has been considered responsible for a late‐onset FD, primarily characterized by a cardiac hypertrophic phenotype [26]. The present study, regarding 13 hemizygous males and 31 heterozygous females, confirmed the causative role of this variant, as it was significantly associated with the FD phenotype, reflected by the FDF score (Figure 1), and with all related clinical domains except cerebrovascular events, which were not associated with any of the pathogenic variants considered collectively (Figure S2A–F). Among the numerical variables investigated, an increased uACR ratio, but not eGFR, was found to be associated with this variant (Tables 2A and 2B).

### 4.6 Strengths and Limitations of the Study

The two main strengths of the study are represented by: (i) the very large number of genotyped subjects, more than 469 thousand subjects of the UK Biobank, representing one of the largest databases of gene variants connected to a large collection of phenotypic features and (ii) the mentioned unbiased approach of the investigation.

The main limitations of the study are: (i) the relatively small number of subjects carrying the gene variants collected in biobanks in comparison with international disease registries, so that in some cases very large confidence intervals in calculations prevented from drawing meaningful conclusions, (ii) the lack of data on LysoGb3 accumulation in plasma or tissues to confirm the diagnosis of FD, not available in the UK Biobank. Consequently, we could assess associations between *GLA* variants and FD‐like phenotypes but not confirm true FD diagnoses. Nonetheless, hemizygous males with pathogenic variants do not require biopsy confirmation in presence of a definite FD phenotype, representing the “true” cases of the present study, and (iii) the rate of missing data in some clinical domains, as the pain domain, that determined an underestimation of the final FDF score, since the lowest class of score was inferred for missing observation.

In conclusion, in the UK Biobank cohort of more than 469 thousand subjects genotyped for *GLA* variants, no significant excess of FD features was found in patients with *GLA* VUS or variants with conflicting interpretation of significance, as p.Asp313Tyr, p.Ala143Thr, p.Ser126Gly and p.Arg118Cys. The p.Asn215Ser variant was confirmed to be associated with the late‐onset form of FD, exhibiting both cardiac and renal features. All non‐pathogenic *GLA* variants exhibited a FD like phenotype only in the presence of an excess of CV risk (age, diabetes, hypertension, dyslipidemia, obesity, smoking). This present study is the first of this kind suggesting that these variants should not be considered causative of FD.

## Author Contributions

**Antonella Giammanco and Carola Maria Gagliardo:** writing – original draft preparation. **Chiara Scrimali, Federica Brucato, and Teresa Maria Grazia Fasciana:** investigation. **Maurizio Averna:** conceptualization. **Angelo Baldassare Cefalu:** supervision, writing – review ± editing. **Davide Noto:** conceptualization, methodology, formal analyses, software, project administration. **Davide Noto:** corresponding author, guarantor for the article.

## Conflicts of Interest

The authors declare no conflicts of interest.

## Supporting information


**Data S1:** Supporting Information

## Data Availability

This research has been conducted using data from UK Biobank, a major biomedical database, and it is part of the project 205 197. The data that support the findings of this study are available from the corresponding author upon reasonable request.
