# Supplementary material for: Clinical and Biochemical Characterization of Fabry Disease Associated GLA Gene Variants: Data From a Large Cohort of 469 Thousand Genotyped Subjects of the UK Biobank Database
Source: J Inherit Metab Dis. 2025 Oct 19;48(6):e70103. doi: 10.1002/jimd.70103 (PMC12535849; doi:10.1002/jimd.70103)
Supplement: Supplementary file 1 — Data S1: Supporting Information [file JIMD-48-0-s001.docx]

# Supplemental Table 1: List of the investigated *GLA* variants.

| **ProteinChange** | **Position** | **Frequency (Hemi/Het/WT)*** | **Ref Base** | **Alt Base** | **Sequence Ontology** | **cDNA change** |
| --- | --- | --- | --- | --- | --- | --- |
| **Conflicting interpretation of pathogenicity** | | | | | | |
| p.Ala368Pro_C | X:101397997 | 10 / 38 / 469447 | C | G | missense_variant | c.1102G>C |
| p.Ala368Thr_C | X:101397997 | 3 / 15 / 469477 | C | T | missense_variant | c.1102G>A |
| p.Arg363His_C | X:101398011 | 0 / 2 / 469477 | C | T | missense_variant | c.1088G>A |
| p.Arg356Gln_C | X:101398032 | 1 / 6 / 469501 | C | T | missense_variant | c.1067G>A |
| p.Ala352Gly_C | X:101398044 | 1 / 1 / 469502 | G | C | missense_variant | c.1055C>G |
| p.Pro343Leu_C | X:101398071 | 3 / 2 / 469499 | G | A | missense_variant | c.1028C>T |
| p.Leu331Phe_C | X:101398378 | 1 / 1 / 468820 | G | A | missense_variant | c.991C>T |
| p.Asp315=_C | X:101398424 | 76 / 195 / 469189 | G | A | synonymous_variant | c.945C>T |
| p.Ile289Val_C | X:101398504 | 0 / 2 / 469436 | T | C | missense_variant | c.865A>G |
| p.Ser238=_C | X:101398872 | 1 / 1 / 469226 | A | G | synonymous_variant | c.714T>C |
| p.Tyr207His_C | X:101400686 | 2 / 1 / 469179 | A | G | missense_variant | c.619T>C |
| p.Ile198Thr_C | X:101400712 | 1 / 4 / 469074 | A | G | missense_variant | c.593T>C |
| p.Asp175Glu_C | X:101401654 | 35 / 76 / 469376 | G | C | missense_variant | c.525C>G |
| p.Asp175Glu_U | X:101401654 | 2 / 2 / 469483 | G | C | missense_variant | c.525C>G |
| p.Ala143Thr_C | X:101401752 | 116 / 317 / 469065 | C | T | missense_variant | c.427G>A |
| p.Ser126Gly_C | X:101401803 | 167 / 328 / 468994 | T | C | missense_variant | c.376A>G |
| p.Arg118Cys_C | X:101403828 | 172 / 441 / 468861 | G | A | missense_variant | c.352C>T |
| p.Gln107Arg_C | X:101403860 | 12 / 14 / 469485 | T | C | missense_variant | c.320A>G |
| p.Asp83Asn_C | X:101403933 | 11 / 31 / 469429 | C | T | missense_variant | c.247G>A |
| p.Gly80Asp_C | X:101403941 | 2 / 1 / 469470 | C | T | missense_variant | c.239G>A |
| p.Glu66Gly_C | X:101403983 | 0 / 1 / 469455 | T | C | missense_variant | c.197A>G |
| p.Glu66Gln_C | X:101403984 | 0 / 2 / 469489 | C | G | missense_variant | c.196G>C |
| p.Thr41=_C | X:101407781 | 66 / 142 / 469317 | G | A | synonymous_variant | c.123C>T |
| p.Trp24Arg_C | X:101407834 | 0 / 2 / 469433 | A | T | missense_variant | c.70T>A |
| p.Leu16=_C | X:101407856 | 72 / 216 / 469254 | A | C | synonymous_variant | c.48T>G |
| p.His9Arg_C | X:101407878 | 0 / 1 / 469455 | T | C | missense_variant | c.26A>G |
| p.Leu3Pro_C | X:101407896 | 7 / 38 / 469438 | A | G | missense_variant | c.8T>C |
| p.Asp313Tyr_C | X:101398432 | 859 / 1990 / 466689 | C | A | missense_variant | c.937G>T |
| **Pathogenic** |  |  |  |  |  |  |
| p.Ile359Thr_P | X:101398023 | 0 / 1 / 469452 | A | G | missense_variant | c.1076T>C |
| p.Arg356Trp_P | X:101398033 | 1 / 1 / 469455 | G | A | missense_variant | c.1066C>T |
| p.Arg301Gln_P | X:101398467 | 0 / 4 / 469387 | C | T | missense_variant | c.902G>A |
| p.Lys240GlufsTer9 | X:101398867 | 0 / 1 / 469226 | TT | - | frameshit truncation | c.718_719del |
| p.Arg227Ter_P | X:101398907 | 0 / 1 / 469365 | G | A | stop_gained | c.679C>T |
| p.Asn215Ser_P | X:101398942 | 13 / 31 / 469312 | T | C | missense_variant | c.644A>G |
| p.Arg363Cys_P | X:101398012 | 6 / 5 / 469480 | G | A | missense_variant | c.1087C>T |
| p.Ile232Thr_P | X:101398891 | 0 / 3 / 469328 | A | G | missense_variant | c.695T>C |
| p.Arg112His_P | X:101403845 | 4 / 6 / 469511 | C | T | missense_variant | c.335G>A |
| **Uncertain significance** | | | | | | |
| p.Leu429LysfsTer? | X:101397810 | 1 / 0 / 467357 | TAAAG | - | frameshit truncation | c.1285_1289del |
| p.Val413Ala_U | X:101397861 | 0 / 2 / 469498 | A | G | missense_variant | c.1238T>C |
| p.Trp399Ser_U | X:101397903 | 4 / 6 / 469273 | C | G | missense_variant | c.1196G>C |
| p.Glu398Gln_U | X:101397907 | 0 / 3 / 469447 | C | G | missense_variant | c.1192G>C |
| p.Gly395Ala_U | X:101397915 | 0 / 4 / 469479 | C | G | missense_variant | c.1184G>C |
| p.Ala381Val_U | X:101397957 | 0 / 1 / 469521 | G | A | missense_variant | c.1142C>T |
| p.Ala370Val_U | X:101397990 | 0 / 1 / 469490 | G | A | missense_variant | c.1109C>T |
| p.Tyr365Asn_U | X:101398006 | 3 / 2 / 469517 | A | T | missense_variant | c.1093T>A |
| p.Met353Ile_U | X:101398040 | 0 / 1 / 469539 | C | T | missense_variant | c.1059G>A |
| p.Val339Ala_U | X:101398083 | 0 / 1 / 469487 | A | G | missense_variant | c.1016T>C |
| p.Pro323Leu_U | X:101398401 | 0 / 2 / 469477 | G | A | missense_variant | c.968C>T |
| p.Val316Ile_U | X:101398423 | 5 / 5 / 469458 | C | T | missense_variant | c.946G>A |
| p.Ala309Val_U | X:101398443 | 0 / 1 / 469452 | G | A | missense_variant | c.926C>T |
| p.Pro305Leu_U | X:101398455 | 1 / 1 / 469287 | G | A | missense_variant | c.914C>T |
| p.Pro305Ser_U | X:101398456 | 1 / 1 / 469361 | G | A | missense_variant | c.913C>T |
| p.Ser304Thr_U | X:101398458 | 0 / 1 / 469445 | C | G | missense_variant | c.911G>C |
| p.Trp277Cys_U | X:101398538 | 40 / 82 / 469374 | C | G | missense_variant | c.831G>C |
| p.Asp264Asn_U | X:101398796 | 2 / 1 / 469435 | C | T | missense_variant | c.790G>A |
| p.Glu251Gly_U | X:101398834 | 0 / 1 / 469347 | T | C | missense_variant | c.752A>G |
| p.Ile242Val_U | X:101398862 | 0 / 1 / 469433 | T | C | missense_variant | c.724A>G |
| p.Arg220Gln_U | X:101398927 | 1 / 1 / 469459 | C | T | missense_variant | c.659G>A |
| p.Pro214Leu_U | X:101398945 | 1 / 1 / 469324 | G | A | missense_variant | c.641C>T |
| p.Ser197Asn_U | X:101400715 | 7 / 2 / 469258 | C | T | missense_variant | c.590G>A |
| p.Asp182Asn_U | X:101401635 | 8 / 23 / 469411 | C | T | missense_variant | c.544G>A |
| p.Val164Ile_U | X:101401689 | 4 / 2 / 469477 | C | T | missense_variant | c.490G>A |
| p.Asp161Asn_U | X:101401698 | 0 / 2 / 469545 | C | T | missense_variant | c.481G>A |
| p.Asp153Asn_U | X:101401722 | 1 / 4 / 469439 | C | T | missense_variant | c.457G>A |
| p.Tyr123Cys_U | X:101403812 | 1 / 3 / 469446 | T | C | missense_variant | c.368A>G |
| p.Arg118His_U | X:101403827 | 1 / 4 / 469433 | C | T | missense_variant | c.353G>A |
| p.Leu106Phe_U | X:101403864 | 2 / 1 / 469503 | G | A | missense_variant | c.316C>T |
| p.Arg105Gly_U | X:101403867 | 0 / 1 / 469534 | T | C | missense_variant | c.313A>G |
| p.Asp101Asn_U | X:101403879 | 0 / 2 / 469516 | C | T | missense_variant | c.301G>A |
| p.Glu87Lys_U | X:101403921 | 2 / 1 / 469445 | C | T | missense_variant | c.259G>A |
| p.Glu71Gly_U | X:101403968 | 0 / 2 / 469473 | T | C | missense_variant | c.212A>G |
| p.Asp61Asn_U | X:101407723 | 3 / 3 / 469483 | C | T | missense_variant | c.181G>A |
| p.Gln57Arg_U | X:101407734 | 0 / 1 / 469510 | T | C | missense_variant | c.170A>G |
| p.Thr41Ser_U | X:101407782 | 1 / 2 / 469522 | G | C | missense_variant | c.122C>G |
| p.Ala29Thr_U | X:101407819 | 0 / 1 / 469499 | C | T | missense_variant | c.85G>A |
| p.Asp25Gly_U | X:101407830 | 0 / 2 / 469536 | T | C | missense_variant | c.74A>G |
| p.Ala15Val_U | X:101407860 | 0 / 1 / 469523 | G | A | missense_variant | c.44C>T |
| p.Ala13Thr_U | X:101407867 | 0 / 1 / 469542 | C | T | missense_variant | c.37G>A |
| p.Cys12Phe_U | X:101407869 | 8 / 12 / 469521 | C | A | missense_variant | c.35G>T |
| p.Asn5Asp_U | X:101407891 | 0 / 2 / 469531 | T | C | missense_variant | c.13A>G |

*Hemi = Male Hemizygous or Female Hemizygous (excluded from calculations), Het = Female Heterozygous. WT = Wild Type. The suffix _C, _P and _U indicate Conflicting, Pathogenic and Uncertain variants.

**Supplemental Table 2: Composition of the Fastex Derived Fabry Score, and correspondence of items between FASTEX and FDF Score**

| **FASTEX** | **FDF Score** |
| --- | --- |
| Nervous system score |  |
| Score Pain |  |
| 0 None | 0 |
| 1 Mild without treatment | NA |
| 2 Moderate without treatment | NA |
| 3 Present and controlled with therapy | 3 |
| 4 Present and not controlled with therapy | NA |
|  |  |
| Score Events |  |
| 0 None | 0 |
| 1 Hyperintensity of white matter | NA |
| 2 TIA | 2 |
| 3 ischaemic or haemorrhagic | 3 |
| 4 Recurrent TIA or stroke | NA |
|  |  |
|  |  |
| Renal system score |  |
| Score Albuminuria (ACR)/proteinuria (PCR) |  |
| 0 ACR <22 mg/g (or <2.5 mg/mmol) | 0 |
| 1 ACR 22–299 mg/g (or 2.5–29 g/mmol) | 1 |
| 2 PCR >300 ≤ 499 mg/g | 2 |
| 3 PCR >500 ≤ 799 mg/g | 3 |
| 4 PCR >800 mg/g | 4 |
|  |  |
| Score eGFR |  |
| 0 <135 mL/min >90 mL/min | 0 |
| 1 >135 mL/min (Hyper filtration) | 1 |
| 2 <90–≥60 mL/min | 2 |
| 3 ≤59–≥30 mL/min | 3 |
| 4 ≤29 mL/min | 4 |
|  |  |
| Cardiac system score |  |
| Score LVH |  |
| 0 No LVH | 0 |
| 1 Diastolic dysfunction | NA |
| 2 Mild LVH (11.5–13.5 mm) | NA |
| 3 Moderate LVH (>13.5–15 mm) or Fibrosis MRI | 3 |
| 4 Severe LVH (>15 mm) | NA |
|  |  |
| Score ECG/arrhythmia Score NYHA |  |
| None 0 | 0 |
| 1 Short PQ, ST alteration 1 I | 1 Short PQ |
| 2 LVH on ECG 2 II | NA |
| 3 AVB, PSVT, AF, NSVT, bradycardia 3 III | 3 |
| 4 PM, ICD 4 | NA |

**Supplemental Table 3: CV risk score model by multiple regression analysis**

| parameters | Estimate | Std. Error | t | pvalue |
| --- | --- | --- | --- | --- |
| (Intercept) | -1.4676379 | 0.0173048 | -84.811 | <2.00E-16 |
| Gender | 0.1018967 | 0.0048037 | 21.212 | <2.00E-16 |
| Age | 0.0479145 | 0.0003093 | 154.89 | <2.00E-16 |
| Diabetes mellitus | 0.2676415 | 0.008739 | 30.626 | <2.00E-16 |
| Dyslipidemia | 0.3965686 | 0.0070815 | 56.001 | <2.00E-16 |
| Obesity | 0.3143449 | 0.0090979 | 34.552 | <2.00E-16 |
| Smoking | 0.0126674 | 0.0048046 | 2.637 | <0.00838 |
| Hypertension | 0.5674794 | 0.0058861 | 96.41 | <2.00E-16 |

**Supplemental Figure 1: Uk Biobank database variables size according to gender**


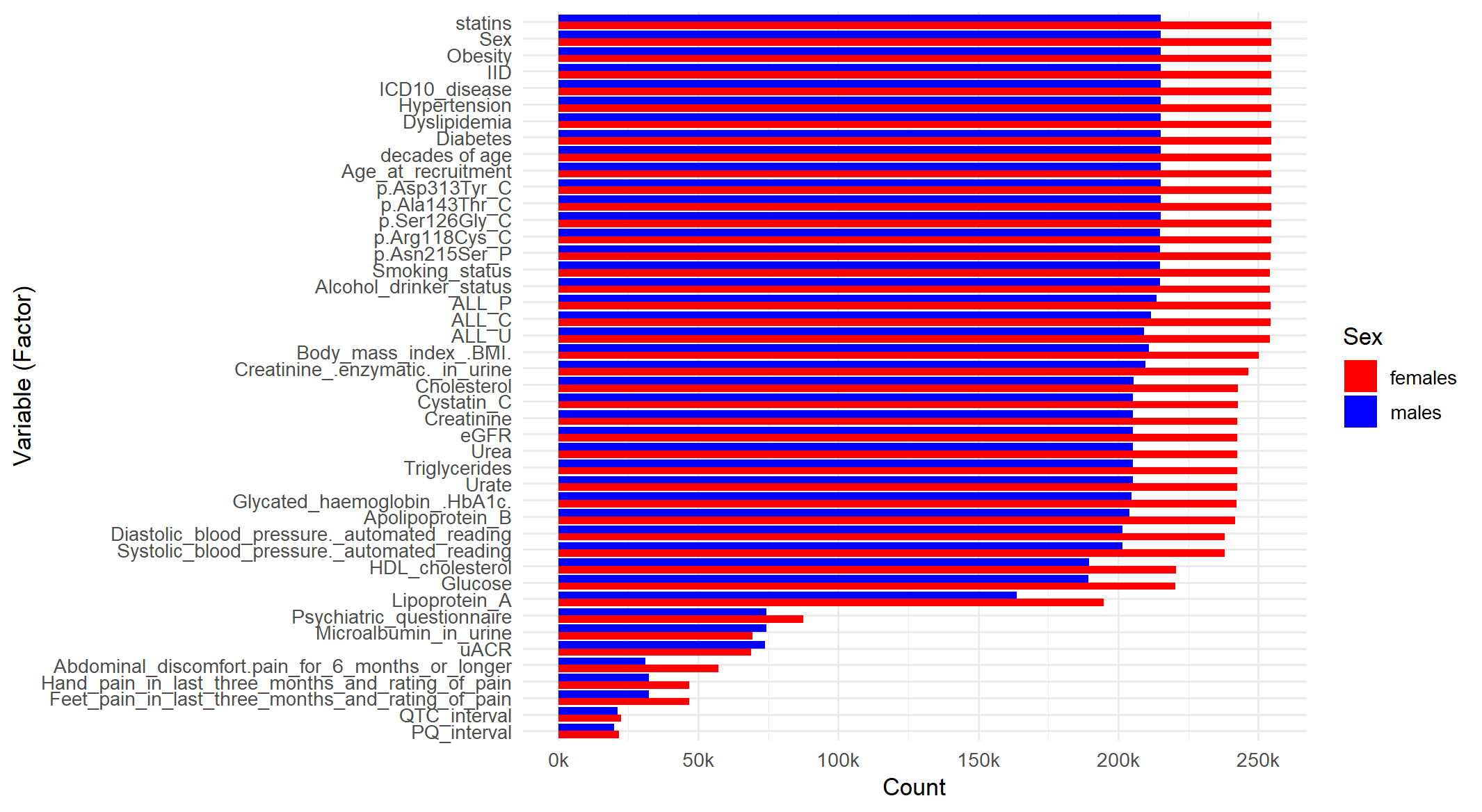


Number of subjects (male and female) available for every variable used for calculation. Missing values were inferred as the lower value (=0) for FDF scoring system.

### Supplemental Figure 2A: Association between *GLA* variants and FDF item, Arrhytmias.

Data are expressed as Odds Ratio and 97.5% confidence intervals in brackets. OR were adjusted for CV risk factors: age, diabetes mellitus, dyslipidemia, hypertension, smoking, obesity. The suffixes in individual variants are : _C = variants with conflicting interpretation of pathogenicity, _P = pathogenic variant. Idicators are proportional to the number of carriers (log transformed).

### Supplemental Figure 2B: Association between *GLA* variants and FDF item, Cardiomyopathy.

Data are expressed as Odds Ratio and 97.5% confidence intervals in brackets. OR were adjusted for CV risk factors: age, diabetes mellitus, dyslipidemia, hypertension, smoking, obesity. The suffixes in individual variants are : _C = variants with conflicting interpretation of pathogenicity, _P = pathogenic variant. Idicators are proportional to the number of carriers (log transformed).

### Supplemental Figure 2C: Association between *GLA* variants and FDF item,

### Albumin/Protein ratio (uACR).

Data are expressed as Odds Ratio and 97.5% confidence intervals in brackets. OR were adjusted for CV risk factors: age, diabetes mellitus, dyslipidemia, hypertension, smoking, obesity. The suffixes in individual variants are : _C = variants with conflicting interpretation of pathogenicity, _P = pathogenic variant. Idicators are proportional to the number of carriers (log transformed).

### Supplemental Figure 2D: Association between *GLA* variants and FDF item,

### Albumin/Protein ratio (uACR).

Data are expressed as Odds Ratio and 97.5% confidence intervals in brackets. OR were adjusted for CV risk factors: age, diabetes mellitus, dyslipidemia, hypertension, smoking, obesity. The suffixes in individual variants are : _C = variants with conflicting interpretation of pathogenicity, _P = pathogenic variant. Idicators are proportional to the number of carriers (log transformed).

### Supplemental Figure 2E: Association between *GLA* variants and FDF item,

### Estimated glomerular filtration rate (eGFR).

Data are expressed as Odds Ratio and 97.5% confidence intervals in brackets. OR were adjusted for CV risk factors: age, diabetes mellitus, dyslipidemia, hypertension, smoking, obesity. The suffixes in individual variants are : _C = variants with conflicting interpretation of pathogenicity, _P = pathogenic variant. Idicators are proportional to the number of carriers (log transformed).

### Supplemental Figure 2F: Association between GLA variants and FDF item,

### Cerebrovascular events.

Data are expressed as Odds Ratio and 97.5% confidence intervals in brackets. OR were adjusted for CV risk factors: age, diabetes mellitus, dyslipidemia, hypertension, smoking, obesity. The suffixes in individual variants are : _C = variants with conflicting interpretation of pathogenicity, _P = pathogenic variant. Idicators are proportional to the number of carriers (log transformed). NA = not enough affected subjects for calculations.

**Supplemental Figure 3: ROC curve analysis of the CV risk score as predictor of the FDF score > 4.**

Roc curve: power of the CV risk score to predict the FD phenotype, represented as FDF score > 4. ROC performance parameters in the figure inset.
